# Mothers’ reports of the difficulties that their children experience in taking methotrexate for Juvenile Idiopathic Arthritis and how these impact on quality of life

**DOI:** 10.1186/1546-0096-11-23

**Published:** 2013-05-28

**Authors:** Kathleen Mulligan, Laura Kassoumeri, Angela Etheridge, Halima Moncrieffe, Lucy R Wedderburn, Stanton Newman

**Affiliations:** 1 School of Health Sciences, City University London, Northampton Square, London, EC1V OHB, UK; 2 Rheumatology Unit, UCL Institute of Child Health, 30 Guilford Street, London, WC1N 1EH, UK; 3 East London NHS Foundation Trust, EastONE, 22 Commercial Street, London, E1 6LP, UK

**Keywords:** Juvenile idiopathic arthritis, Methotrexate, Side effects, Intolerance, Anticipatory nausea, Psychological, Quality of life

## Abstract

**Background:**

Children who take methotrexate for juvenile idiopathic arthritis may experience side effects, including nausea and vomiting, leading to anticipatory nausea in some children, and fear of injections or blood tests. The aim of this study was to examine the prevalence and extent of these difficulties and their impact on quality of life.

**Methods:**

Participants were mothers of children with JIA who were currently taking methotrexate (MTX). Mothers completed a questionnaire about MTX that was developed for the study, two questions from the treatment subscale of the Pediatric Quality of Life Inventory (PedsQL) Rheumatology scale to assess needle-related problems and the Child Health Questionnaire 50-item parent version (CHQ-PF50) to assess health-related quality of life (HRQoL).

**Results:**

171 mothers participated in the study. More than half of children were reported to have experienced one or more of: nausea or vomiting after taking MTX, anticipatory nausea, fear of blood tests or fear of injections. There was no significant difference in reported rates of sickness or needle-related problems between MTX responders (ACR70 or above), partial responders (ACR30 or ACR50) and non-responders. In multivariate analyses, variables that were significant independent predictors of one or more MTX-related difficulties included younger age, taking MTX subcutaneously and having a larger number of currently active joints. Feeling sick after taking MTX was a significant independent predictor of poorer scores on the physical summary scale of the CHQ-PF50. Anxiety about injections and feeling sick after taking MTX were significant independent predictors of poorer scores on the psychosocial summary scale.

**Conclusions:**

Difficulties in taking MTX are experienced by a significant proportion of children with JIA and these may have an adverse impact on HRQoL. Approaches to help minimize these difficulties are required.

## Background

Methotrexate (MTX) is the first choice disease-modifying antirheumatic drug (DMARD) for the treatment of Juvenile Idiopathic Arthritis (JIA) [1,2]. Along with proven efficacy in reducing arthritis, both physical and psychosocial quality of life (QoL) have also been found to improve in children with JIA treated with MTX [3]. Notwithstanding these benefits, some children experience difficulties related to taking MTX. The most common recognised side effects are gastrointestinal, including nausea and vomiting [4,5]. The use of folic acid or folinic acid has been suggested to reduce such side effects [6], although little formal evidence base for this is yet available. If a child experiences nausea or vomiting after taking MTX, anticipatory nausea (symptoms occurring before medication is taken), and reluctance to take MTX may develop [5,7]. Further problems can arise as a result of fear of injections or the monthly monitoring blood tests that are necessary while taking MTX.

Although these MTX-related problems are well recognised [7], the extent to which they may impact on adherence to the medication and on the child’s quality of life is less clear. We are not aware of any study that has systematically examined the relationship between MTX-related problems and quality of life. As parents are key facilitators in their child’s medication regime [8], it is also important to gain an insight into parents’ views of MTX and whether any difficulties their child experiences in taking MTX affects their willingness to allow their child to try other medications that may be prescribed in the future. The Sport Aiding Medical Research for Kids (SPARKS) Childhood Arthritis Response to Medication Study (CHARMS) [9,10] aims to define the factors that influence the response of children with JIA to methotrexate. As part of the study we investigated the difficulties arising from the use of MTX for JIA. This paper reports data collected from mothers of children with JIA about the extent of MTX-related difficulties experienced by their children and factors associated with these difficulties. We also examined how MTX-related difficulties impact on reported adherence, the children’s quality of life and mothers’ views about MTX. Finally, we examined whether there was any difference between responders and non-responders [11] in MTX-related difficulties or mothers’ views about MTX.

## Methods

Participants were recruited as part of the SPARKS Childhood Arthritis Response to Medication Study (SPARKS-CHARMS), which examines genetic, immunobiological and psychological aspects of response to MTX or anti-TNF therapy given for arthritis [9,10,12,13]. The study recruits children of any age with juvenile idiopathic arthritis (JIA), defined by ILAR criteria [14], who are under the care of the Rheumatology service at Great Ormond Street Hospital for Children (GOSH) or the Adolescent Rheumatology service at University College Hospital, London, UK. The practice in these two centres is that children start on a MTX dose equivalent to 15 mg per metre squared (as per evidence of efficacy [15]). In those who respond very well, the dose may not be increased as the child grows i.e. actual dose falls; in some children, the dose is increased with growth to maintain the 15 mg per metre square dose. The practice in these two centres is to offer folic acid from the start to all children taking MTX (1mg per day as syrup or in some cases 5mg per week). The practice for nausea is to offer and encourage use of anti-emetics, typically Ondansetron, to be used pre and post MTX as required. The practice with route is that for those who start on oral and develop regular nausea, many are offered the change to subcutaneous.

Children were recruited to the main study if they: a) were about to start taking MTX or anti-TNF, b) were currently taking MTX or, c) had taken MTX in the past.

For the psychological part of the study, we aimed to recruit the parent(s) of 230 children. Parents whose children were aged up to sixteen years, plus children aged five to sixteen years were asked to complete questionnaires about experiences of taking MTX and HRQoL. Recruitment was restricted to English-speakers because the questionnaire was available only in English (this was not a restriction in the main study). Parents were given the questionnaires to complete in the clinic but they could choose to take them home and return them by post. The aim was to recruit at least one parent but if both parents attended the clinic they were both invited to participate. Although fathers and the patients themselves participated in the study, mothers were the largest respondent group. We have previously shown that there can be high levels of discordance between mothers’ and fathers’ views about their child’s JIA [9], and others have reported discordance between parent and child reports [16], so to help ensure consistency we have limited this analysis to reports from one parent (mothers). This analysis is restricted to mothers of those children in the study who had taken MTX for at least six months, and were still taking it at the time the questionnaires were completed.

The study had full ethical approval from the Institute of Child Health/GOSH Local Research Ethics Committee, and all participants gave full, informed written consent (parental consent and age appropriate child/young person assent). The study conforms to the principles outlined in the Declaration of Helsinki.

### Study questionnaires

A questionnaire on the experience of taking MTX was developed for the study as there was no appropriate questionnaire available on this topic. The questionnaire asked how frequently the child a) felt sick before taking MTX; b) felt sick after taking MTX; c) vomited after taking MTX. Response options were: never/hardly ever, less than once a month, about once a month, two or three times a month, every week. The questionnaire also asked about how frequently a dose of MTX was missed (1 item), and mothers’ views of MTX (4 items). The experience of needle-related problems was assessed with two questions from the treatment subscale of the Pediatric Quality of Life Inventory (PedsQL) Rheumatology scale [17]. The questions asked how much of a problem anxiety about injections and about blood tests have been in the past month. Response options were: never, almost never, sometimes, often, almost always.

Adherence was estimated by the reported frequency a dose of MTX was missed for any reason. Response options were: never/hardly ever, less than once a month, about once a month, two or three times a month, every week. Missing a dose about once a month or more frequently was classified as non-adherence. This cut-off was used as it would represent an adherence rate of almost 80%, which is a conventional cut-off used in adherence research [18].

To capture their views on taking other medications, mothers were also asked how willing they would be for their child to try other arthritis medications prescribed in the future. This was assessed with a 100mm visual analogue scale (VAS) from ‘not willing’ (0) to ‘very willing’ (100).

Health-related quality of life (HRQoL) was assessed with the British version of the Child Health Questionnaire 50-item parent version (CHQ-PF50) [19], which has been validated for use in children with JIA [20]. This measure provides two standardised norm-based summary scores – a physical summary score (PhS) and psychosocial summary score (PsS) – which each range from 0 – 100, have a mean of 50 and standard deviation of 10. Higher scores indicate a better quality of life.

Data on child’s age, gender, JIA category disease duration, MTX route, duration of use and dose, and MTX responder status, (assessed using the core set criteria and internationally agreed definition of improvement [11]) were also collected. Current disease severity was assessed by the number of limited and inflamed joints. Data on mother’s age and education were collected.

### Statistical analysis

Statistical analyses were conducted in SPSS Statistics 21. To examine potential risk factors for MTX problems we examined the association between occurrence of each problem (sickness before taking, sickness after taking, vomiting after taking, anxiety about injections, anxiety about blood tests) with age, gender, current disease severity, duration of MTX use, route of MTX administration (oral or subcutaneous), current MTX dose and whether or not the child was taking folic acid, using binary logistic regression analyses. A problem was categorised as being present if it occurred frequently i.e. response options two or three times a month/every week (sickness-related questions) or often/almost always (needle-related problems).

The potential impact of MTX-related difficulties on adherence was analysed using Fishers Exact Tests to compare those classified as adherent or non-adherent on their likelihood of having experienced problems.

To examine which of the MTX experiences accounted for most variance in HRQoL measured with the CHQ-PF50, the independent variables were included in hierarchical multiple regressions using stepwise method. Two regressions were performed: one analysis examined the physical and the other the psychosocial summary scores of the CHQ-PF50. The independent variables were entered into the regression in blocks in the following order: 1. demographic variables; 2. disease variables; 3. route by which MTX taken, duration of MTX use, current dose; 4. MTX problems. This order was used because it enables examination to be made as to whether experience of MTX added to the explanation of quality of life once disease severity had been taken into account.

To examine whether experience of MTX-related problems affected mothers’ ratings of MTX and willingness to allow their child to take other medications, t-tests were conducted comparing those who rarely/never experienced each problem with those who had experienced it. To examine the relationship between current disease severity and mothers’ ratings of MTX, Spearman rho correlations were conducted.

To compare responders, partial responders and non-responders on experience of MTX-related difficulties and mothers’ views of MTX, we conducted χ^2^ tests and analyses of variance (ANOVA) respectively.

## Results

**Figure 1:**
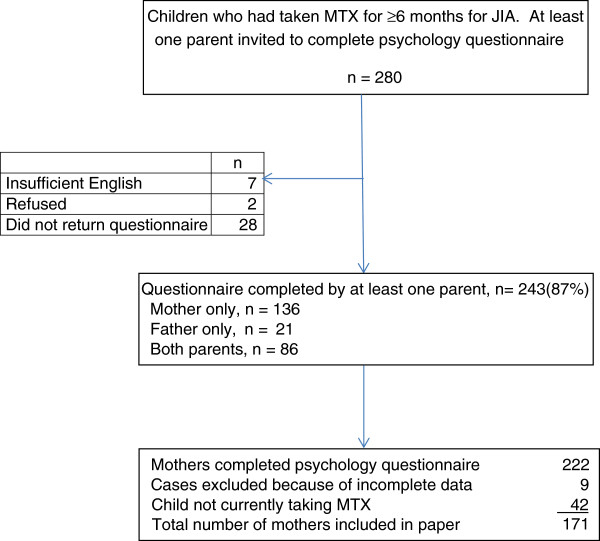
Recruitment flowchart.

A recruitment flowchart is shown in Figure 1. The parents of 280 children who had taken MTX for ≥6 months were approached to take part in the psychological part of the study. Of these, a questionnaire was completed by at least one parent of 243 (87%) children. Mothers of 222 children completed questionnaires but 9 were excluded from this analysis because of large amounts of missing data. A further 42 are not included in this analysis because their child was not taking MTX at the time the questionnaire was completed. Data for this paper were thus provided by 171 mothers. Demographic data of the 171 children and their mothers are shown in Table 1. As expected in JIA, the children were predominantly female (71.9%). The distribution among JIA categories was also as expected for use of MTX in JIA, with polyarticular rheumatoid factor negative (RF-) JIA being the largest group (36.8%) and extended oligoarticular the next largest (24.6%). Of those for whom response to MTX [11] was known, 50.4% were classified as good responders, that is they achieved improvement at ACR70 level or better, which is also in line with expectations.

**Table 1:** Sample characteristics of patients and respondents

**Children’s characteristics**	
n	171
Gender, n (%) female	123 (71.9)
Age in years when questionnaire data completed, mean (S.D.)	9.0 (4.1)
JIA category, n (%)	
*Systemic*	26 (15.2)
*Oligo persistent*	13 (7.6)
*Oligo extended*	42 (24.6)
*Poly RF –*	63 (36.8)
*Poly RF +*	9 (5.3)
*Poly, RF status unknown*	2 (1.2)
*Psoriatic*	6 (3.5)
*ERA*	10 (5.8)
Disease duration in years, mean (S.D.) (n=169)	4.0 (3.3)
Responder status (known for n = 117)	
*Non-responder*	34 (29.1)
*ACR30*	83 (70.9)
*ACR50*	74 (63.2)
*ACR70*	59 (50.4)
Current disease severity, median, range, (IQR)	
*Number of active joints (known for n = 158)*	0, 0–12, 0-1
*Number of limited joints (known for n = 155)*	0, 0–32, 0-2
**Mothers’ characteristics**	
Age in years, mean (S.D.)	38.5 (6.7)
Education*, n (%)	
≤GCSE or equivalent	105 (63.2)
Advanced level or equivalent	23 (13.8)
Degree/Postgraduate	38 (22.9)
*Not stated*	5

*Advanced level – national examinations usually taken at age 18, required for university admission.

GCSE – national examinations usually taken at age 16.

The majority of children (n = 100, 58.5%) were currently taking MTX subcutaneously but most of these (n = 76) had previously taken it orally. Table 2 shows the numbers who have moved from oral administration to subcutaneous and vice versa during their time taking MTX.

**Table 2:** Methotrexate history of study patients

Age in years at starting MTX, mean (S.D.) (n=168)	6.1 (3.9)
Duration in months of MTX use, median (IQR)	29.0 (11.5 – 60.5)
Current MTX dose (mg/m^2^/week), median (IQR)	12.5 (10 – 15)
MTX route, n (%)	
*Oral route throughout*	55 (32.2)
*Current route oral but previously subcutaneous*	16 (9.4)
*Subcutaneous route throughout*	24 (14.0)
*Current route subcutaneous but previously oral*	76 (44.4)

### Extent of methotrexate-related problems

**Figure 2:**
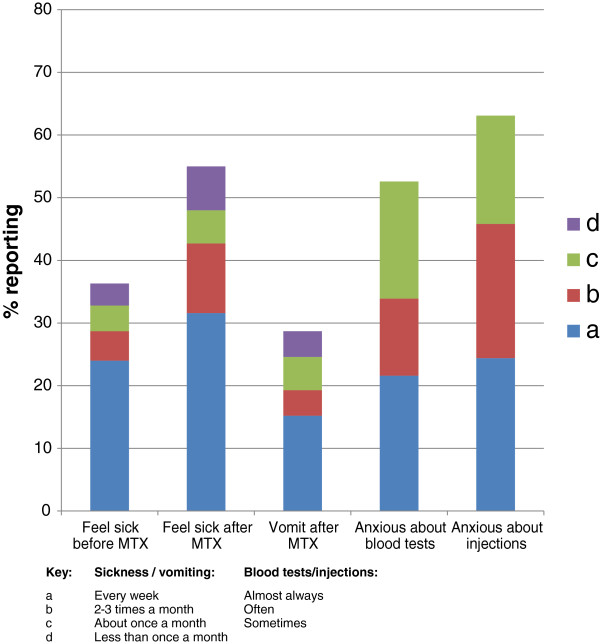
**Frequency of mothers’ reports of their children**^**'**^**s methotrexate-related sickness and needle-related fear.**

The extent of MTX-related problems, including nausea and/or vomiting, before or after the weekly MTX treatment, and fear of needles, is shown in Figure 2. More than half of the children were reported to have experienced one or more of these difficulties. Almost a third of children were reported to feel sick every week after taking MTX and almost a quarter experienced weekly anticipatory nausea. Fifteen per cent of children were reported to have vomited every week after taking MTX. Fear of injections and/or blood tests rated as often or almost always was reported in over a third of children with over half reported as experiencing them at least sometimes.

### Potential ‘risk factors’ for methotrexate-related problems

**Figure 3:**
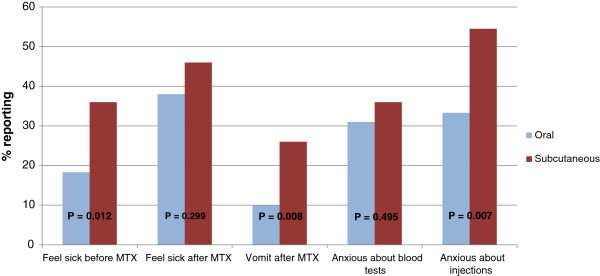
Comparison between oral and subcutaneous administration on experience of MTX-related difficulties.

Univariate and multivariate logistic regression analyses of factors associated with MTX-related problems are shown in Table 3. A variety of parameters were associated with MTX-related problems. In univariate analyses, feeling sick before or after taking MTX was significantly related to: older age, larger current MTX dose and longer duration of taking MTX. Subcutaneous route and higher current disease activity also significantly increased risk of feeling feeling sick before MTX. Vomiting after taking MTX was significantly related to subcutaneous route and longer duration of taking MTX. Anxiety about blood tests was significantly related to younger age and shorter duration of MTX use. Anxiety about injections was significantly related to younger age, subcutaneous route and higher current disease activity. When these risk factors were simultaneously entered into logistic regression models, age, MTX route and current disease activity remained significant independent predictors of one or more of the problems. Fear of blood tests and injections decreased with age. Children who were taking MTX subcutaneously were more likely to vomit after taking it than children who were taking MTX orally (see also Figure 3). Anxiety about injections was also more common in those taking MTX subcutaneously. Those with a larger number of currently active joints were more likely to feel sick before taking MTX and to be anxious about injections. Gender, number of limited joints and whether or not the child was taking folic acid were not related to any of the MTX-related difficulties in univariate or multivariate analyses.

**Table 3:** Multivariate logistic regression models of risk factors for MTX-related difficulties

**Potential risk factors**		**MTX problems**									
		**Feel sick before taking MTX**		**Feel sick after taking MTX**		**Vomit after taking MTX**		**Anxious about blood tests**		**Anxious about injections**	
	**n**	**Unadjusted Exp(β) (95% CI)**	**Adjusted Exp (β) † (95% CI)**	**Unadjusted Exp(β) (95% CI)**	**Adjusted Exp (β) † (95% CI)**	**Unadjusted Exp(β) (95% CI)**	**Adjusted Exp (β) † (95% CI)**	**Unadjusted Exp(β) (95% CI)**	**Adjusted Exp (β) † (95% CI)**	**Unadjusted Exp(β) (95% CI)**	**Adjusted Exp (β) † (95% CI)**
Age	171	1.10 (1.01–1.19)	1.06 (0.92–1.22)	1.15 (1.06–1.24)	1.11 (0.98–1.25)	1.06 (0.97–1.17)	1.07 (0.92–1.24)	0.82 (0.75–0.90)	**0.76 (0.65-0.89)**	0.85 (0.79–0.93)	**0.83 (0.73–0.95)**
Sex											
Male	48	1.00	1.00	1.00	1.00	1.00	1.00	1.00	1.00	1.00	1.00
Female	123	0.56	0.89	1.06	1.38	0.52	0.48	1.18	0.87	0.79	0.78
		(0.28–1.15)	(0.37–2.16)	(0.54–2.08)	(0.62–3.09)	(0.24–1.16)	(0.19–1.25)	(0.58–2.42)	(0.35-2.18)	(0.40–1.55)	(0.32–1.88)
MTX current route											
Oral	71	1.00	1.00	1.00	1.00	1.00	**1.00**	1.00	1.00	1.00	**1.00**
Subcutaneous	100	2.51	2.33	1.39	1.50	3.21	**3.75**	1.25	0.79	2.40	**2.31**
		(1.21–5.19)	(0.96–5.66)	(0.75–2.58)	(0.69–3.26)	(1.31–7.89)	**(1.28–11.06)**	(0.66–2.40)	(0.34-1.87)	(1.27–4.54)	**(1.02–5.22)**
MTX current dose (mg)	171	1.10	1.06	1.11	1.06	1.05	0.98	0.95	1.10	0.96	1.03
		(1.02–1.19)	(0.95–1.19)	(1.04–1.20)	(0.96–1.17)	(0.97–1.14)	(0.88–1.10)	(0.88–1.02)	(0.97-1.25)	(0.89–1.02)	(0.93–1.15)
Duration of MTX use in months	168	1.02	1.01	1.01	1.01	1.02	1.01	0.98	0.996	0.995	1.00
		(1.01 - 1.03)	(0.99-1.03)	(1.00- 1.03)	(0.99-1.02)	(1.00 -1.03)	(0.99-1.03)	(0.97-0.997)	(0.98-1.01)	(0.98 - 1.01)	(0.98 - 1.01)
Taking folic acid											
No	24	1.00	1.00	1.00	1.00	1.00	1.00	1.00	1.00	1.00	1.00
Yes	124	0.41	0.55	0.48	0.68	0.41	0.57	1.02	0.98	0.76	0.42
		(0.17 – 1.01)	(0.19 – 1.58)	(0.20 – 1.17)	(0.25 – 1.85)	(0.16 – 1.08)	(0.18 – 1.76)	(0.41 – 2.59)	(0.32 - 3.03)	(0.27 – 1.60)	(0.14 – 1.25)
Current disease severity:											
No. active joints	158	1.24	**1.29**	1.07	1.09	1.07	0.91	1.20	1.15	1.23	**1.40**
		(1.06 – 1.44)	**(1.05 – 1.60)**	(0.92 – 1.23)	(0.91 – 1.30)	(0.91 – 1.25)	(0.74 – 1.13)	(0.97 – 1.29)	(0.95 - 1.40)	(1.03 – 1.46)	**(1.09 – 1.79)**
No. limited joints	155	1.09	1.01	1.01	0.95	1.09	1.04	0.97	0.97	0.98	0.93
		(0.99 – 1.20)	(0.91 – 1.12)	(0.92 – 1.10)	(0.85 – 1.06)	(0.99 – 1.21)	(0.92 – 1.17)	(0.88 – 1.08)	(0.85 - 1.10)	(0.90 – 1.07)	(0.82 – 1.06)

†Adjusted for all variables simultaneously.

Exp(β) – predicted change in odds for a one unit increase in the predictor variable.

Statistically significant findings in the adjusted analyses are shown in bold.

### The relationship between methotrexate experiences and adherence

The rate of reported adherence was very high, with only seven children (4.1%) classified as non-adherent. Fisher’s Exact Tests showed no significant differences in adherence between those who had MTX-related problems and those who did not.

### Mothers’ views about methotrexate and about treatment in general

In response to the questions, ‘How do you rate methotrexate overall?’ and ‘Are you satisfied with the effects of methotrexate?’, mothers’ ratings were fairly high with 111/168 (66.1%) rating it as very good or excellent and 130/170 (76.5%) being mostly or completely satisfied with its effects (Additional file 1: Table S1).

A comparison of mothers’ ratings of MTX between those with and without MTX-related difficulties is shown in Additional file 2: Table S2. Mothers’ rating of the severity of side effects compared to their expectations was worse in those who reported sickness before or after or vomiting after taking MTX. No differences were found between those reporting or not reporting MTX-related difficulties in mothers’ ratings of effectiveness, satisfaction with effects, feeling their child received the right medication for them, overall rating of MTX, or willingness to allow their child to take future medications.

There was a small but statistically significant negative correlation between the number of currently active joints (that is, active at the time the questionnaire was completed) and mothers’ satisfaction with MTX. There were also small but statistically significant negative correlations between the number of limited joints and mothers’ rating of the effectiveness of MTX and their overall rating of MTX (Additional file 3: Table S3).

### Relationship with responder status

Responder status [11] after the first 6 months of taking MTX was known for 117 (68.4%) children (Table 1). Core set criteria for response to MTX variables were not always available because data were collected retrospectively, sometimes several years after the child first started taking MTX. Not all children were first prescribed MTX at the study hospital therefore core outcome variables before and six months after taking MTX for the first time were not necessarily available. The duration since first prescribed MTX was significantly longer in those for whom responder status was unknown (mean years 4.0 (S.D. 2.4) v. 1.5 (2.0), t = 6.93, df = 166, p < 0.0005). We did not find any statistically significant differences between responder status known or unknown on age, gender, JIA category, HRQoL or any of the MTX experiences.

In those for whom responder status was known (n = 117), there were no significant differences between responders and partial- or non-responders in reported sickness or needle-related problems or in mothers’ ratings of MTX (Additional file 4: Table S4).

### Relationship between methotrexate-related problems and children’s quality of life

The mean (S.D.) score of 40.4 (14.2) on the physical summary score of the CHQ-PF50 is approximately 1 S.D. below population norms whereas the respective score of 47.3 (12.1) on the psychosocial summary score is closer to the norm.

Multivariate analyses of the relation between experiences of MTX and the physical and psychosocial summary scales of the CHQ-PF50 are shown in Table 4.

**Table 4:** Stepwise multiple regression analysis of variables related to health-related quality of life

**Model**		***B***	**S.E.**	***β***	***P***	***R***^***2***^
*Physical summary score*						
1	age	-.583	.284	-.168	.042	.028
2	age	-.463	.268	-.134	.086	.150
	Number of limited joints	-1.340	.296	-.350	.000	
3	age	-.475	.265	-.137	.075	.176
	Number of limited joints	-1.145	.306	-.299	.000	
	Number of active joints	-1.094	.510	-.170	.034	
4	age	-.586	.263	-.169	.027	.214
	Number of limited joints	-1.058	.302	-.276	.001	
	Number of active joints	-1.064	.500	-.166	.035	
	MTX administration route	-5.691	2.181	-.198	.010	
5	age	-.200	.325	-.058	.539	.235
	Number of limited joints	-1.023	.299	-.267	.001	
	Number of active joints	-1.002	.496	-.156	.045	
	MTX administration route	-4.756	2.210	-.165	.033	
	MTX current weekly dose	-.572	.289	-.185	.050	
6	age	.023	.317	.007	.941	.305
	Number of limited joints	-1.084	.287	-.283	.000	
	Number of active joints	-.874	.476	-.136	.068	
	MTX administration route	-3.892	2.126	-.135	.069	
	MTX current weekly dose	-.495	.277	-.160	.077	
	feel sick after taking MTX	-7.951	2.121	-.278	.000	
*Psychosocial summary score*						
1	feel sick after taking MTX	-5.624	1.976	-.230	.005	.053
2	feel sick after taking MTX	-5.338	1.940	-.218	.007	.097
	anxious about blood tests	-5.347	2.027	-.209	.009	

Variables entered into the models:

Block 1: age, sex.

Block 2: age, sex, number of active joints, number of limited joints.

Block 3: age, sex, number of active joints, number of limited joints, MTX administration route, duration of MTX use, MTX current dose.

Block 4: age, sex, number of active joints, number of limited joints, MTX administration route, duration of MTX use, MTX current dose, feel sick before taking MTX, feel sick after taking MTX, vomit after taking MTX, anxious about blood tests, anxious about injections.

In addition to the number of limited joints, feeling sick after taking MTX was a significant independent predictor of poorer scores on the physical summary scale of the CHQ-PF50, after controlling for demographic variables, disease severity, MTX dose, duration of use and administration method. Anxiety about blood tests and feeling sick after taking MTX were significant independent predictors of poorer scores on the psychosocial summary scale. Overall, variables in the models accounted for 30.5% of variance in the physical summary score and 9.7% of the variance in the psychosocial summary score.

## Discussion

Key findings of this study are that over half of children taking MTX for JIA were reported to experience some difficulty. Reports of nausea before and after taking MTX were more common in children whose method of taking MTX was subcutaneous as opposed to oral. Feeling sick after taking MTX and anxiety about blood tests were independent predictors of poorer quality of life. Finally, in spite of these difficulties, the majority of mothers rated MTX positively overall.

It is well known to clinicians that children who take MTX for JIA may experience the problems reported here [5] but we are aware of only one other study that has systematically documented the extent of these problems [21], however that study did not examine the relationship with quality of life. Bulatović et al. [21] found that just over half of children had ‘MTX intolerance’ so the findings of our current study are consistent with that research. Although the benefits of MTX for treating JIA are well known [2], it is also important to recognise and take all possible steps to minimise such difficulties. These strategies may include anti-emetic medication, and support from specialist nurses or psychologists. A small study of behavioural therapy (systemic desensitization or cognitive behavioural therapy) for children with JIA having nausea suggests that this can be beneficial [7].

Our study found that taking MTX subcutaneously was associated with a greater risk of post-administration vomiting. As the subcutaneous route has been suggested to be advantageous for treatment efficacy of MTX [22,23] new approaches to managing the difficulties in taking MTX subcutaneously could provide an important addition in JIA treatment.

The findings of this study have important clinical implications. The impact of problems in taking MTX on quality of life emphasise that such difficulties have a wider impact, and this highlights the importance of looking for ways of minimising the difficulties and distress in taking MTX. Currently, psychological input is usually reserved for those who report significant problems in dealing with JIA and a pilot study has shown the potential benefits of such an approach [7]. However, many parents may value advice on how to manage the distress their children experience in taking MTX. Anticipatory nausea is a good example of a conditioned response and psychological techniques have a good history of treating such responses [24]. There is a large literature on how to enhance self-management of chronic illness [25-28] and extending this approach to JIA may prove beneficial.

Our findings suggest that for children with JIA, difficulties in taking MTX are not necessarily explained by whether the child’s arthritis does or does not improve as a result of taking MTX. Mothers valued MTX as a treatment and were satisfied with the treatment decisions made in spite of the reported difficulties experienced. This is an important finding as it indicates that mothers appreciated the medication despite the problems experienced, and suggests that their experience is unlikely to have an on-going impact on their attitudes to medication.

There are several limitations to our study. The data presented were collected from proxy respondents, in this case the mothers of children with JIA, and patients and proxy respondents are known to differ in their evaluations [16]. However, approximately 39% of the children were aged under eight years and may have been unable to provide all of the self-report data for the study. We considered it important to be able to examine the experience of taking MTX for young children and caregiver ratings are widely used in assessment in JIA [11]. The study of MTX intolerance by Bulatovic et al. [17] included children between 2 and 18 years of age, therefore proxy respondents were also presumably included in that study.

We did not have information on the responder status of all children because these data were collected retrospectively. However, there were no differences in responses about treatment between those for whom responder status was known and those where it was unknown.

Data about problems related to taking MTX orally or subcutaneously are complicated by the fact that many children may change from oral to subcutaneous administration during the course of taking the medication. This will be the case in any study that includes children who have been taking MTX for some time. It would be of interest for future research to collect information about side effects and perceptions of MTX from the time of first prescription and to monitor how these alter over time and with changes in method of administration. It would also be of interest to examine how the occurrence of other adverse events, for example a raised alanine aminotransferase (ALT) test result, would influence findings; we did not have blood test result data to examine this question. The findings reported here are based on cross-sectional data therefore, as with all such data, we cannot infer causal direction.

## Conclusions

In conclusion, our study suggests that difficulties in taking MTX for children with JIA are common, and not explained simply by the child’s clinical response to the medication. These difficulties can have an adverse effect on the child’s HRQoL and approaches to help minimize and overcome them are required.

## Competing interest

The authors declare that they have no competing interests.

## Authors’ contributions

LRW and SN - study conception and design, analysis and interpretation of data and drafting of the manuscript. KM - acquisition, analysis and interpretation of data and drafting of the manuscript. LK, AE and HM acquisition and interpretation of data and drafting of the manuscript. All authors read and approved the final manuscript.

## Supplementary Material

Additional file 1: Table S1 Mothers’ views about methotrexate. Click here for file

Additional file 2: Table S2 Comparison of means (S.D.) of mothers’ ratings of MTX between those with and without MTX-related difficulties. Click here for file

Additional file 3: Table S3 Spearman rho correlations between current disease severity and mothers’ views about MTX. Click here for file

Additional file 4: Table S4 Comparison between responders, partial-responders and non-responders on their experience of MTX-related difficulties and mothers’ ratings of MTX. Click here for file
