# Supplementary material for: Mothers’ reports of the difficulties that their children experience in taking methotrexate for Juvenile Idiopathic Arthritis and how these impact on quality of life
Source: Pediatr Rheumatol Online J. 2013 May 28;11:23. doi: 10.1186/1546-0096-11-23 (PMC3679741; doi:10.1186/1546-0096-11-23)
Supplement: Additional file 1: Table S1 — Mothers’ views about methotrexate. [file 1546-0096-11-23-S1.pdf]

Additional file 1. Mothers' views about methotrexate

	**n (%)**
Compared to what you expected, how do you rate the effectiveness of methotrexate?	
A lot better than expected	91 (53.2)
A little better than expected	26 (15.2)
About the same as expected	33 (19.3)
A little worse than expected	11 (6.4)
A lot worse than expected	6 (3.5)
Don't know	1 (0.6)
Not stated (NS)	3 (1.8)
Compared to what you expected how do you rate the side effects of methotrexate?	
A lot better than expected	68 (39.8)
A little better than expected	23 (13.5)
About the same as expected	47 (27.5)
A little worse than expected	18 (10.5)
A lot worse than expected	14 (8.2)
NS	1 (0.6)
Are you satisfied with the effects of methotrexate?	
Completely	73 (42.7)
For the most part	57 (33.3)
To some extent	28 (16.4)
Not at all	11 (6.4)
Don't know	1 (0.6)
NS	1 (0.6)
Do you feel that your child received the treatment that was right for them?	
Completely	109 (63.7)
For the most part	45 (26.3)
To some extent	14 (8.2)
Not at all	3 (1.8)
How do you rate methotrexate overall?	
Excellent	53 (31.0)
Very good	58 (33.9)
Good	30 (17.5)
Fair	17 (9.9)
Poor	8 (4.7)
Don't know	2 (1.2)
NS	3 (1.8)
**Future medication:**	
How willing you would be for your child to try other arthritis medications prescribed in the future[a], mean (S.D.)	6.72 2.59)

a. 10cm VAS, higher score = more willing
