# Supplementary material for: Mothers’ reports of the difficulties that their children experience in taking methotrexate for Juvenile Idiopathic Arthritis and how these impact on quality of life
Source: Pediatr Rheumatol Online J. 2013 May 28;11:23. doi: 10.1186/1546-0096-11-23 (PMC3679741; doi:10.1186/1546-0096-11-23)
Supplement: Additional file 2: Table S2 — Comparison of means (S.D.) of mothers’ ratings of MTX between those with and without MTX-related difficulties. [file 1546-0096-11-23-S2.pdf]

Additional file 2. Comparison of means (S.D.) of mothers' ratings of MTX between those with and without MTX-related difficulties.

Rating of MTX:	Feel sick before taking MTX				Feel sick after taking MTX				Vomit after taking MTX				Anxious about blood tests				Anxious about injections			
	No	Yes[1]	t	P	No	Yes[1]	t	P	No	Yes[1]	t	P	No	Yes[2]	t	P	No	Yes[2]	t	P
Effectiveness[a]	4.14 (1.15)	4.06 (1.19)	0.40	0.69	4.18 (1.08)	4.04 (1.26)	0.74	0.46	4.11 (1.14)	4.15 (1.23)	-0.18	0.86	4.09 (1.18)	4.18 (1.11)	-0.47	0.64	4.13 (1.13)	4.08 (1.21)	0.29	0.77
Side effects[a]	3.88 (1.30)	3.14 (1.24)	3.38	0.001	4.19 (1.09)	2.94 (1.27)	6.89	<0.0005	3.93 (1.19)	2.55 (1.25)	5.96	<0.0005	3.64 (1.39)	3.71 (1.17)	-0.30	0.77	3.67 (1.47)	3.61 (1.13)	0.27	0.78
Satisfaction with effects[b]	3.16 (0.91)	3.12 (0.97)	0.22	0.83	3.24 (0.89)	3.01 (0.97)	1.61	0.11	3.13 (0.92)	3.21 (0.96)	-0.45	0.66	3.19 (0.95)	3.07 (0.90)	0.79	0.43	3.21 (0.91)	3.09 (0.96)	0.83	0.41
Feel child received treatment right for them[c]	3.53 (0.70)	3.49 (0.79)	0.35	0.73	3.61 (0.64)	3.40 (0.81)	1.94	0.054	3.51 (0.71)	3.58 (0.79)	-0.49	0.63	3.55 (0.64)	3.47 (0.86)	0.71	0.48	3.56 (0.64)	3.45 (0.82)	0.94	0.35
Overall rating[d]	3.85 (1.16)	3.73 (1.18)	0.61	0.54	3.87 (1.17)	3.74 (1.15)	0.68	0.50	3.84 (1.18)	3.70 (1.08)	0.65	0.52	3.78 (1.16)	3.88 (1.18)	-0.49	0.62	3.88 (1.10)	3.74 (1.26)	0.76	0.45
Willingness to try other medication[e]	6.61 (2.68)	6.99 (2.33)	-0.87)	0.38	6.69 (2.64)	6.75 (2.54)	-0.14	0.89	6.68 (2.63)	6.86 (2.42)	-0.35	0.72	6.70 (2.55)	6.74 (2.68)	-0.08	0.94	6.40 (2.71)	7.14 (2.42)	-1.87	0.06

1 reported to occur 'two or three times a week' / 'every week'
2 reported to have been a problem 'often' / 'almost always' during the past month
a Scale 1 – 5, higher score = better than expected
b Scale 1 – 4, higher score = more satisfied
c Scale 1 – 4, higher score = stronger agreement that child received treatment that was right for them
d Scale 1 – 5, higher score = better
e Scale 0 – 10, higher score = more willing

Statistically significant findings are shaded
