# Supplementary material for: Mothers’ reports of the difficulties that their children experience in taking methotrexate for Juvenile Idiopathic Arthritis and how these impact on quality of life
Source: Pediatr Rheumatol Online J. 2013 May 28;11:23. doi: 10.1186/1546-0096-11-23 (PMC3679741; doi:10.1186/1546-0096-11-23)
Supplement: Additional file 3: Table S3 — Spearman rho correlations between current disease severity and mothers’ views about MTX. [file 1546-0096-11-23-S3.pdf]

Additional file 3. Spearman rho correlations between current disease severity and mothers' views about MTX.

Mothers' ratings of MTX:	Number of active joints	Number of limited joints
Effectiveness	-.105	-.191*
Side effects	.067	-.060
Satisfaction with effects	-.187*	-.151
Feel child received treatment right for them	-.123	-.087
Overall rating	-.151	-.189*
Willingness to try other medication	.063	.085

*$p<0.05$
