# Supplementary material for: Mothers’ reports of the difficulties that their children experience in taking methotrexate for Juvenile Idiopathic Arthritis and how these impact on quality of life
Source: Pediatr Rheumatol Online J. 2013 May 28;11:23. doi: 10.1186/1546-0096-11-23 (PMC3679741; doi:10.1186/1546-0096-11-23)
Supplement: Additional file 4: Table S4 — Comparison between responders, partial-responders and non-responders on their experience of MTX-related difficulties and mothers’ ratings of MTX. [file 1546-0096-11-23-S4.pdf]

Additional file 4.

Comparison between responders, partial-responders and non-responders on their experience of MTX-related difficulties and mothers' ratings of MTX.

| | Responder status | | | | |
|---|---|---|---|---|---|
| | Non-responder (n = 34) | Partial responder (ACR30/50) (n = 24) | Responder (ACR70) (n = 59) | Statistic | P |
| **MTX-related difficulty, n (%) who experienced difficulty:** | | | | | |
| Feel sick before taking MTX[1] | 11 (32.4) | 9 (37.5) | 11 (18.6) | $\chi^2 = 3.96$ | 0.14 |
| Feel sick after taking MTX[1] | 15 (44.1) | 11 (45.8) | 22 (37.3) | $\chi^2 = 0.70$ | 0.70 |
| Vomit after taking MTX[1] | 9 (26.5) | 5 (20.8) | 6 (10.2) | $\chi^2 = 4.34$ | 0.11 |
| Anxious about blood tests[2] | 12 (35.3) | 6 (25.0) | 23 (39.0) | $\chi^2 = 1.47$ | 0.48 |
| Anxious about injections[2] | 16 (47.1) | 8 (34.8) | 28 (48.3) | $\chi^2 = 1.28$ | 0.53 |
| **Rating of MTX, mean (S.D.)** | | | | | |
| Effectiveness[a] | 3.88 (1.27) | 3.96 (1.11) | 4.32 (1.17) | F = 1.67 | 0.19 |
| Side effects[a] | 3.41 (1.26) | 3.75 (1.36) | 3.86 (1.33) | F = 1.27 | 0.28 |
| Satisfaction with effects[b] | 3.00 (1.02) | 2.79 (1.02) | 3.19 (0.89) | F = 1.55 | 0.22 |
| Feel child received treatment right for them[c] | 3.44 (0.89) | 3.38 (0.77) | 3.47 (0.63) | F = 0.15 | 0.86 |
| Overall rating[d] | 3.68 (1.22) | 3.65 (1.43) | 3.88 (1.05) | F = 0.45 | 0.64 |
| Willingness to try other medication[e] | 6.65 (2.31) | 7.43 (1.85) | 6.71 (2.68) | F = 0.90 | 0.41 |

1 reported to occur 'two or three times a week' / 'every week'
2 reported to have been a problem 'often' / 'almost always' during the past month
a Scale 1 – 5, higher score = better than expected
b Scale 1 – 4, higher score = more satisfied
c Scale 1 – 4, higher score = stronger agreement that child received treatment that was right for them
d Scale 1 – 5, higher score = better
e Scale 0 – 10, higher score = more willing
